# Perioperative administration of buffered versus non-buffered crystalloid intravenous fluid to improve outcomes following adult surgical procedures: a Cochrane systematic review

**DOI:** 10.1186/s13741-018-0108-5

**Published:** 2018-12-13

**Authors:** Peter M. Odor, Sohail Bampoe, Ahilanandan Dushianthan, Elliott Bennett-Guerrero, Suzie Cro, Tong J. Gan, Michael P. W. Grocott, Michael F. M. James, Michael G. Mythen, Catherine M. N. O’Malley, Anthony M. Roche, Kathy Rowan, Edward Burdett

**Affiliations:** 1 Department of Anaesthesia and Critical Care, University College London, Gower St, London, WC1E 6BT UK; 2 Centre for Anaesthesia and Perioperative Medicine, University College London, London, UK; 3 General Intensive Care Unit, University Hospital Southampton NHS Foundation Trust, Southampton, UK; 4 Department of Anesthesiology, Stony Brook Medicine, Stony Brook, NY USA; 5 Medical Research Council Clinical Trials Unit, London, UK; 6 Critical Care Group, Clinical and Experimental Sciences, Faculty of Medicine, University of Southampton, Southampton, UK; 7 Department of Anaesthesia, University of Cape Town, Cape Town, South Africa; 8 Department of Anaesthesia, St James’s Hospital, Dublin, Ireland; 9 Department of Anesthesiology and Pain Medicine, University of Washington, Seattle, WA USA; 10 Intensive Care National Audit & Research Centre, London, UK; 11 Department of Anaesthesia, UCL Centre for Anaesthesia, London, UK

**Keywords:** Fluid therapy, Plasma substitutes, Surgery

## Abstract

**Background:**

Buffered intravenous fluid preparations contain substrates to maintain acid-base status. The objective of this systematic review was to compare the effects of buffered and non-buffered fluids administered during the perioperative period on clinical and biochemical outcomes.

**Methods:**

We searched MEDLINE, EMBASE, CINAHL and the Cochrane Library until May 2017 and included all randomised controlled trials that evaluated buffered versus non-buffered fluids, whether crystalloid or colloid, administered to surgical patients. We assessed the selected studies for risk of bias and graded the level of evidence in accordance with Cochrane recommendations.

**Results:**

We identified 19 publications of 18 randomised controlled trials, totalling 1096 participants. Mean difference (MD) in postoperative pH was 0.05 units lower immediately following surgery in the non-buffered group (12 studies of 720 participants; 95% confidence interval (CI) 0.04 to 0.07; *I*^2^ = 61%). This difference did not persist on postoperative day 1. Serum chloride concentration was higher in the non-buffered group at the end of surgery (10 trials of 530 participants; MD 6.77 mmol/L, 95% CI 3.38 to 10.17). This effect persisted until postoperative day 1 (5 trials of 258 participants; MD 8.48 mmol/L, 95% CI 1.08 to 15.88). Quality of this evidence was moderate. We identified variable protocols for fluid administration and total volumes of fluid administered to patients intraoperatively. Outcome data was variably reported at disparate time points and with heterogeneous patient groups. Consequently, the effect size and overall confidence interval was reduced, despite the relatively low inherent risk of bias. There was insufficient evidence on the effect of fluid composition on mortality and organ dysfunction. Confidence intervals of this outcome were wide and the quality of evidence was low (3 trials of 276 participants for mortality; odds ratio (OR) 1.85, 95% CI 0.37 to 9.33; *I*^2^ = 0%).

**Conclusions:**

Small effect sizes for biochemical outcomes and lack of correlated clinical follow-up data mean that robust conclusions on major morbidity and mortality associated with buffered versus non-buffered perioperative fluid choices are still lacking. Buffered fluid may have biochemical benefits, including a significant reduction in postoperative hyperchloraemia and metabolic acidosis.

**Electronic supplementary material:**

The online version of this article (10.1186/s13741-018-0108-5) contains supplementary material, which is available to authorized users.

## Background

Major surgery presents an important threat to internal homeostasis of fluid and electrolytes, partly due to the volume depleting effects of haemorrhage, evaporative loss and pre-operative dehydration, or by excessive fluid administration causing oedema and organ dysfunction. Intravenous fluid solutions used for resuscitation and maintenance purposes should support the circulation adequately to replace missing plasma, whilst avoiding metabolic disturbance or other adverse effects. Modern perioperative fluid management is based on the principle that, as an intervention with risks and benefits, fluids should only be provided to affect a meaningful clinical variable. This principle influences decisions made for monitoring requirements, timing of administration, volume dosing of fluids and the type of fluids provided to patients.

Intravenous fluids, whether crystalloid or colloid, can be categorised as buffered or non-buffered. Hartmann’s fluid formulation more closely matches the constituents of human plasma than 0.9% saline, containing a physiological buffer that helps to maintain acid-base balance. The composition of Hartmann’s fluid also includes additional electrolytes found in plasma, including potassium, magnesium and calcium. Provision of buffered crystalloid fluids may have benefits over 0.9% saline, in which the electrolyte composition is significantly different to the plasma that it is intended to replace.

The primary objective of this systematic review is to investigate the clinical effects of perioperative administration of buffered fluids, such as Hartmann’s solution, when compared with non-buffered fluids administered during all types of surgery.

## Methods

This paper is an abridged version of a previously published Cochrane systematic review (Bampoe et al. 2017). We prepared this manuscript according to guidelines published by Cochrane (Higgins and Green 2011) and the PRISMA statement for systematic reviews and meta-analysis (Moher et al. 2009). The full systematic review protocol is available in the original Cochrane review (Bampoe et al. 2017).

### Search strategy

We searched publications in the Cochrane Central Register of Controlled Trials (CENTRAL; 2016), MEDLINE (1966 to May 2017), Embase (1980 to May 2017) and the Cumulative Index to Nursing and Allied Health Literature (CINAHL; 1982 to May 2017). No language restrictions were applied to the search criteria. Relevant conference abstract proceedings were also searched. Forward and backward citation tracking of all identified studies was performed. The full search strategy used can be found in the review protocol (Bampoe et al. 2017) and in Additional file 1.

### Inclusion and exclusion criteria

We included randomised controlled trials (RCTs) in which patients received intravenous fluids with and without a buffer (bicarbonate or bicarbonate precursor buffer, such as maleate, gluconate, lactate, or acetate) for the purpose of plasma volume expansion or maintenance during the perioperative period. To minimise confounding factors, we considered only RCTs in which the sole difference between experimental and control arms involved the presence or absence of an electrolyte buffer in the fluid. We excluded studies that compared crystalloids with colloids and those that compared fluids with different colloid components. However, we included trials with three or more arms that satisfied the other inclusion criteria. The perioperative period was defined as extending from 2 h before the start of surgery up to 6 h after surgery or until arrival to a post-anaesthetic care unit. We included only studies that used isotonic fluids (osmolarity 250 to 350 mmol/L) and a broadly physiological concentration of sodium (120 to 160 mmol/L).

### Data collection and analysis

Five review authors (TG, EB, AR, SB and PO) independently screened titles and abstracts of search results to remove irrelevant studies. Two review authors (SB and PO) then reviewed full texts of potentially relevant titles and identified studies that matched inclusion criteria. Data on study characteristics and outcomes were independently extracted from eligible studies by two authors (SB and PO) with disagreements resolved by consensus or by consultation with a third review author (EB). We contacted the authors of included trials to request required data missing from published manuscripts.

### Primary outcomes

1. Mortality (all time frames reported)

### Secondary outcomes

1. Clinically significant organ system dysfunction (including renal, pulmonary, hepatic, gastrointestinal, coagulation and central nervous system)
2. Surrogate measures of organ system dysfunction including urine output, serum creatinine, partial pressure of arterial carbon dioxide (PaCO_2_), nausea, and vomiting
3. Biochemical or electrolyte disturbances including pH, base excess, and serum bicarbonate, sodium, potassium, calcium and chloride
4. Serum measures of coagulation such as prothrombin time, activated partial thromboplastin time, von Willebrand factor, antithrombin 3 activity, fibrinogen and thromboelastography
5. Blood loss or transfusion requirement
6. Postoperative hospital length of stay
7. Functional health status and quality of life measures
8. Cost

### Assessment of risk of bias

We used the Cochrane risk of bias tool to assess the quality of study design and extent of potential bias and considered the following domains: sequence generation, allocation concealment, blinding of participants, personnel and outcome assessors, incomplete data and selective outcome reporting (Higgins and Green 2011).

### Statistical analysis

Data were analysed using Review Manager, version 5.3 (The Nordic Cochrane Centre 2012). For continuous measures (e.g. urine output, serum electrolytes, post-operative pH) we calculated mean differences (MD) with 95% confidence intervals (CIs) using an inverse variance method. For dichotomous outcomes (e.g. mortality, organ system failure), we calculated odds ratios (OR) with 95% CI, using the Mantel-Haenszel method for common outcomes (> 5%) and Peto OR for rare outcomes (< 5%). When studies included more than two groups, we merged data into groups when the intervention was equivalent. Some studies included groups of participants who did not receive the interventions of interest, and we excluded these groups from analyses.

We conducted meta-analysis when it was reasonable to assume that studies were estimating the same underlying treatment effect. We quantified the degree of heterogeneity in trial results using the *I*^2^ statistic (Higgins and Green 2011). We assumed significant heterogeneity when *I*^2^ was ≥ 40%. When heterogeneity was significant, we used random-effects models. When *I*^2^ was < 40%, we used a fixed effect model for analysis.

We planned to perform subgroup analysis to explore sources of heterogeneity between studies. This was not possible because we found insufficient studies reporting our anticipated primary outcome of mortality. We performed sensitivity analysis for the primary outcome to explore the robustness of results based upon variation in study quality and risk of bias assessment.

We judged the quality of evidence using GRADE (GRADE Working Group, McMaster University 2015; Guyatt et al. 2011). We based our assessment of the quality of evidence on assessments of imprecision, inconsistency, risk of bias, and indirectness for all studies reporting specific outcome measures. We considered the starting point to be “high quality” because of the randomised design of all included studies. We downgraded quality by one or two levels on the basis of assessment of GRADE criteria and assessment of the methodological quality and design of included studies.

## Results

### Study selection and characteristics

**Fig. 1:**
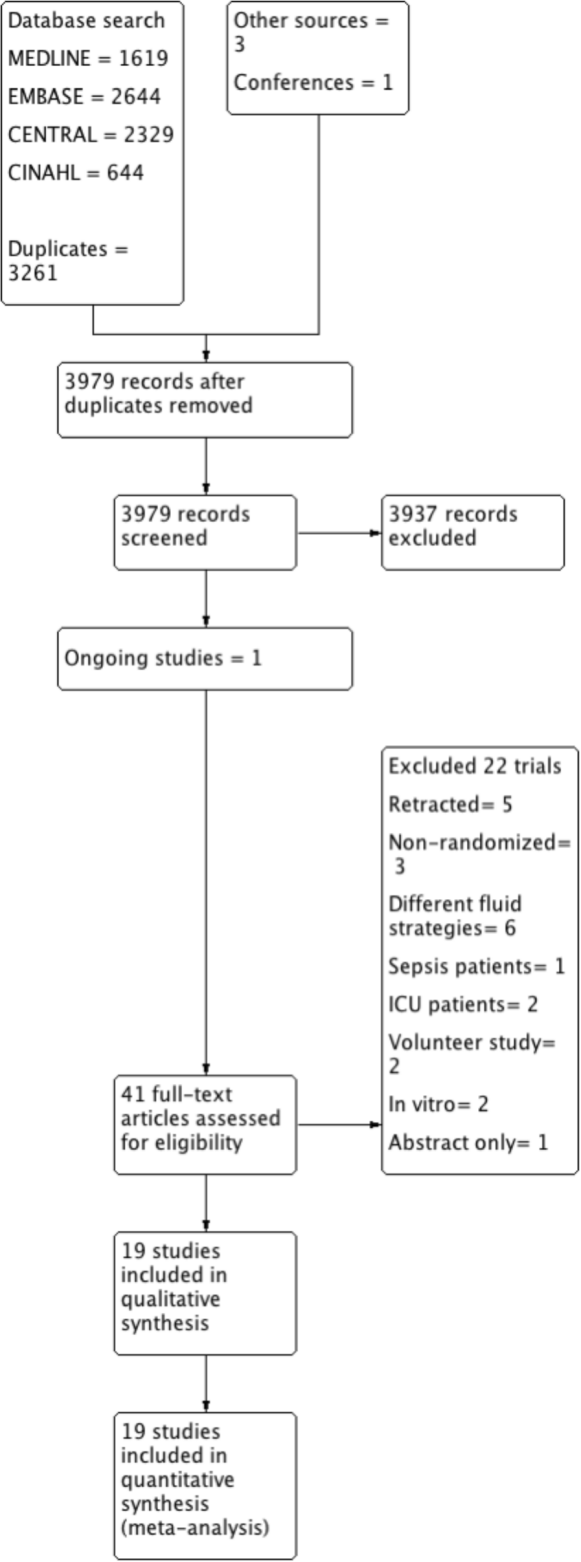
Prisma study flow diagram

We identified 3979 unique citations from database searches, manual searches and citation reviews. After screening by title and abstract we then retrieved 41 full-text manuscripts for further analysis. Following the review, 19 publications of 18 RCTs met the inclusion criteria for study design, participants and interventions. These 18 RCTs were incorporated into the quantitative and qualitative analysis stage. Five trials were rejected because of subsequent retractions of the paper, five trials did not incorporate buffered fluid strategies, five trials were of non-surgical patients, two were in vitro studies and one trial was only available in abstract form. The PRISMA flow chart is provided in Fig. 1.

The eighteen RCTs included a total of 1096 participants, of whom 563 received buffered fluids and 533 received non-buffered fluids. Two papers reported one trial, but different outcomes were described in the two separate papers, showing no overlap, so these publications were considered separately (Martin et al. 2002; Moretti et al. 2003). Five studies included patients with renal transplants (Hadimioglu et al. 2008; Khajavi et al. 2008; Kim et al. 2013; Nuraei et al. 2010; O’Malley et al. 2005). As this population was different from the population undergoing other perioperative procedures, we performed sensitivity analysis, when possible, for renal outcomes such as intraoperative urine output.

Most studies were small, single-site investigations. Only two trials had a sample size of over 100 patients across both intervention and control groups. Alongside the five studies of renal transplant surgery, major elective surgery constituted the majority of procedures. This included major orthopaedic, vascular (open aortic aneurysm repair), gastrointestinal, hepatobiliary, gynaecological and urological surgery. One study of patients undergoing coronary artery bypass grafting was included and two studies included patients with minor surgical procedures, which did not involve an invasion of body cavities. No studies of patients undergoing emergency surgery were identified.

The exact fluid type used and protocol for fluid delivery to participants varied between studies. Of 18 included trials, 13 used crystalloids in both their experimental and control arms. Of these studies, nine compared lactated Ringer’s solution with 0.9% saline and four compared Plasmalyte 148 with 0.9% saline. The remaining six publications of five trials used colloid solutions in their experimental and control arms, comparing a buffered hydroxyethyl starch (HES) solution versus a non-buffered HES solution. High molecular weight (MW) HES was used in four RCTs (Martin et al. 2002; Moretti et al. 2003; Gan et al. 1999; Wilkes et al. 2001), and two used low MW HES (Kulla et al. 2008; Base et al. 2011).

Exclusive use of only buffered and non-buffered fluids in each group was only maintained in seven of the 18 trials. All other studies reported overlap, with the administration of a combination of buffered and non-buffered fluids in the control arm of the study. Hence, most studies actually compared a partially buffered fluid regimen versus a totally buffered fluid regimen.

### Risk of bias in included studies

All trials were randomised, with a total of 16 studies that referred to blinding or double-blinding in their design and had a low risk of performance and detection bias. Fifteen trials provided details about allocation sequence generation and twelve studies described allocation concealment; these studies were considered to be at low risk of selection bias. We judged only one trial to be at high risk of attrition bias because a high proportion of participants dropped out of the trial owing to the administration of non-protocol intravenous fluids; the remainder were a low or unclear risk of bias. We did not detect reporting bias and therefore categorised all studies as low risk.

Pharmaceutical companies that manufactured an intervention of interest funded five of the included studies (Martin et al. 2002; Moretti et al. 2003; Gan et al. 1999; Wilkes et al. 2001; Base et al. 2011). Although each study clearly disclosed these funding sources, we considered these studies to be at unclear risk of bias. Two other studies did not report sufficient detail about outcomes of interest, and we therefore considered them to be at unclear risk of bias (Kulla et al. 2008; Heidari et al. 2011).

**Fig. 2:**
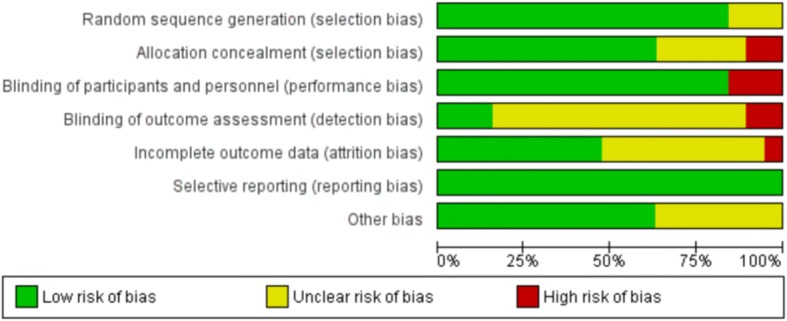
Risk of bias graph. Review authors’ judgments about each risk of bias item presented as percentages across all included studies

Generally, participant numbers in these trials were low, with four trials enrolling fewer than 20 participants in each arm. Consequently, many outcome measures are reported in small group sizes, reducing overall confidence in effect size, despite relatively low inherent bias in the included studies. Risk of bias of included studies is summarised in Fig. 2.

### Primary outcome

**Fig. 3:**
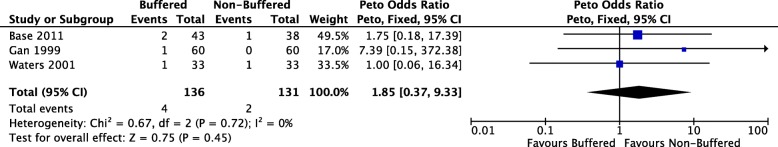
Comparison: buffered perioperative fluids vs non-buffered fluids. Outcome: mortality

Three clinical trials with a total of 267 participants reported mortality (Gan et al. 1999; Base et al. 2011; Waters et al. 2001). Mortality was low in both groups: 2.9% (4/136) in the buffered group and 1.5% (2/131) in the non-buffered group. The limited data suggests no significant mortality differences between groups (OR 1.85, 95% CI 0.37 to 9.33; *I*^2^ = 0%). The quality of evidence was downgraded from high to low owing to the imprecision of trial results due to small sample sizes, wide confidence intervals and methodological variability between studies. Studies reporting mortality presented few events, and all three studies were considered to be at unclear risk of bias for this outcome assessment. Overall confidence in the effect estimate is low. A forest plot for the primary outcome is provided in Fig. 3.

### Secondary outcomes

#### Clinically significant organ system dysfunction

We found low-quality and insufficient evidence to support any effects of fluid therapies on postoperative organ failure. Renal failure leading to the requirement for renal replacement therapy was reported in four trials (Hadimioglu et al. 2008; Kim et al. 2013; O’Malley et al. 2005; Waters et al. 2001), although three of these studies included participants with the confounding effect of pre-existing organ insufficiency (i.e. participants undergoing renal transplant for renal failure) (Hadimioglu et al. 2008; Kim et al. 2013; O’Malley et al. 2005). There was no evidence to support a lower risk of renal failure with buffered fluids (OR 0.82, 95% CI 0.34 to 1.98; *I*^2^ = 0%). A single study reported respiratory failure, enrolling 81 participants with a 9.3% (4/43) incidence of post-operative respiratory failure in the buffered group and 2.6% (1/38) in the non-buffered group. No reports of cardiac, hepatic, gastrointestinal or neurological failure were recorded as outcomes measures.

#### Surrogate measures of organ system dysfunction

##### Urine output

Eight trials with a total of 459 participants reported urine output during the intraoperative period and on the first postoperative day (Kim et al. 2013; O’Malley et al. 2005; Kulla et al. 2008; Gan et al. 1999; Wilkes et al. 2001; Waters et al. 2001; Scheingraber et al. 1999; Takil et al. 2002). Mean urine output reported intraoperatively was 872 ml for the buffered fluid group and 799 ml for the non-buffered fluid group. The mean difference was 6.1 ml higher in the buffered group (95% CI − 128.41 to 140.61; *I*^2^ = 49%). Sensitivity analysis was performed to exclude the four studies that included renal transplant patients (Khajavi et al. 2008; Kim et al. 2013; Nuraei et al. 2010; O’Malley et al. 2005), which confirmed no important differences between groups for intraoperative urine output. One of the two trials reporting urine output on the first post-operative day enrolled renal transplant patients and reported disproportionately large volumes of urine output (Hadimioglu et al. 2008). Due to this clinical heterogeneity, no further meta-analysis was conducted on this outcome measure.

##### Post-operative serum creatinine

Two trials of 113 participants reported relative post-operative serum creatinine change at two time points: immediately post-operatively and on post-operative day one (Wilkes et al. 2001; Waters et al. 2001). No groups showed any significant differences. Mean difference was 6.96 μmol/L lower in the buffered group (95% CI − 27.42 to 13.50; *I*^2^ = 89%) in the immediate post-operative measurement and 4.94 μmol/L lower in the non-buffered group (95% CI -5.91 to 15.78; *I*^2^ = 12%) on post-operative day one.

Absolute creatinine was reported at time points from post-operative day one to day seven. Three trials with a total of 235 participants reported absolute immediate post-operative creatinine values (Nuraei et al. 2010; Kulla et al. 2008; Waters et al. 2001). The mean creatinine in two studies of non-renal transplant participants was 76.72 μmol/L in the buffered fluid group and 79.53 μmol/L in the non-buffered group. One study included renal transplant patients, reporting a significantly different mean creatinine as 530 μmol/L in the buffered fluid group and 460 μmol/L in the non-buffered group (MD 70 μmol/L higher; 95% CI 14.31 to 125.69). Data show no important overall differences between groups. Overall, the MD was − 1.31 μmol/L lower in the non-buffered group (95% CI − 9.30 to 6.68; *I*^2^ = 71%).

Three trials with a total of 211 participants reported postoperative day one creatinine (Hadimioglu et al. 2008; Kim et al. 2013; Kulla et al. 2008). Two studies enrolled renal transplant patients (Hadimioglu et al. 2008; Kim et al. 2013). Overall data show a mean difference 6.26 μmol/L lower in the buffered group (95% CI − 21.17 to 8.64; *I*^2^ = 0%); there were no significant differences between subgroups of renal transplant and non-transplant patients.

Four trials of participants undergoing renal transplants reported post-operative day 3 and day 7 creatinine (Hadimioglu et al. 2008; Khajavi et al. 2008; Nuraei et al. 2010; O’Malley et al. 2005). There was no significant difference in creatinine between the buffered group and non-buffered groups at either time point.

In summary, only one trial showed a significant difference in creatinine outcomes (Nuraei et al. 2010), with a higher immediately post-operative creatinine measured in the buffered fluid group. There were no other statistically (or clinically) significant differences.

##### Partial pressure of arterial carbon dioxide (PaCO_2_)

Seven trials with a total of 446 participants reported postoperative PaCO_2_ at two time points (Hadimioglu et al. 2008; Kim et al. 2013; Nuraei et al. 2010; Kulla et al. 2008; Wilkes et al. 2001; Song et al. 2015; Takil et al. 2002. Results show a statistically (but unlikely clinically) significant higher mean PaCO_2_ of 35.0 mmHg in the buffered fluid group than in the non-buffered fluid group (MD 1.05 mmHg, 95% CI 0.15 to 1.94; *I*^2^ = 0%). Two trials with a total of 91 participants reported postoperative day one PaCO_2_ of 41 mmHg in the buffered fluid group and 37.7 mmHg in the non-buffered fluid group (Kulla et al. 2008; Takil et al. 2002). PaCO_2_ was significantly higher in the buffered group (MD 3.3 mmHg, 95% CI 2.03 to 4.64; *I*^2^ = 0%).

##### Postoperative vomiting

Three trials reported 21/84 (25%) episodes of postoperative vomiting in the buffered fluid group and 28/84 (33%) episodes of postoperative vomiting in the non-buffered fluid group (Moretti et al. 2003; Wilkes et al. 2001; Heidari et al. 2011). There was no significant difference in post-operative vomiting between groups (OR 0.66, 95% CI 0.34 to 1.30; *I*^2^ = 20%).

#### Biochemical or electrolyte disturbance

##### pH

**Fig. 4:**
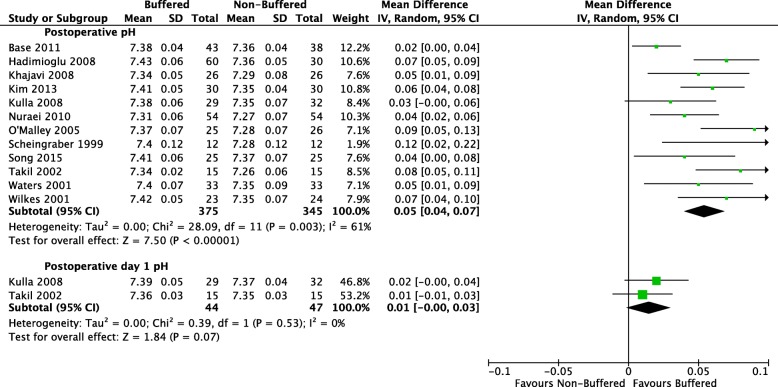
Comparison: buffered perioperative fluids vs non-buffered fluids. Outcome: postoperative pH

Twelve studies with a total of 720 participants reported postoperative pH (Hadimioglu et al. 2008; Khajavi et al. 2008; Kim et al. 2013; Nuraei et al. 2010; O’Malley et al. 2005; Kulla et al. 2008; Wilkes et al. 2001; Waters et al. 2001; Scheingraber et al. 1999; Song et al. 2015; Takil et al. 2002). Reporting was heterogeneous with different time intervals. Mean postoperative pH was 7.38 in the buffered fluid group and 7.32 in the non-buffered fluid group. There was a significant difference, with postoperative pH 0.05 units lower (95% CI − 0.04 to − 0.07; I^2^ = 61%) in the non-buffered group. A forest plot of this outcome is found in Fig. 4.

##### Base excess

**Fig. 5:**
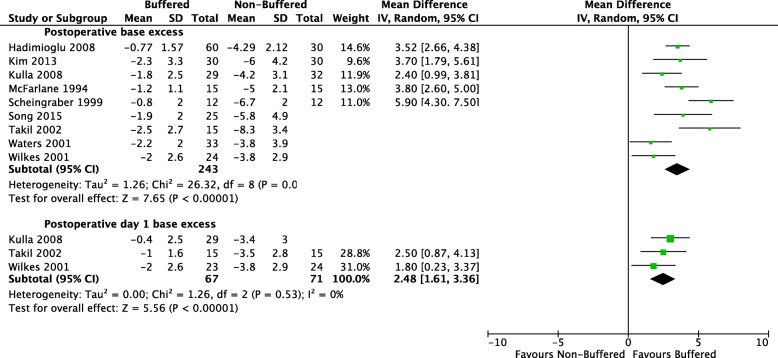
Comparison: buffered perioperative fluids vs non-buffered fluids. Outcome: post-operative base excess

Investigators reported this outcome at various time intervals. Nine studies with a total of 459 participants reported postoperative base excess. Mean base excess was negative for both fluid groups: − 1.65 mmol/L in the buffered fluid group and − 5.02 mmol/L in the non-buffered fluid group (Hadimioglu et al. 2008; Kim et al. 2013; O’Malley et al. 2005; Wilkes et al. 2001; Waters et al. 2001; Scheingraber et al. 1999; Song et al. 2015; Takil et al. 2002; McFarlane and Lee 1994). There was a significant difference between groups, with postoperative base excess 3.51 mmol/L lower in the non-buffered fluid group than in the buffered fluid group (95% CI 2.61 to 4.41). A forest plot of this outcome is found in Fig. 5.

##### Serum bicarbonate

Seven studies with a total of 478 participants reported postoperative serum bicarbonate. Mean postoperative serum bicarbonates was 21.6 mmol/L in the buffered fluid group and 18.6 mmol/L in the non-buffered fluid group (Hadimioglu et al. 2008; Kim et al. 2013; O’Malley et al. 2005; Waters et al. 2001; Scheingraber et al. 1999; Song et al. 2015; Takil et al. 2002). There was a significant difference between groups, with serum bicarbonate 3.14 mmol/L lower in the non-buffered group (95% CI 2.30 to 3.98).

##### Serum sodium

Eight trials with a total of 447 participants reported a postoperative serum sodium concentration of 137.3 mmol/L in the buffered fluid group and 139.4 mmol/L in the non-buffered fluid group (Khajavi et al. 2008; Kim et al. 2013; Nuraei et al. 2010; Kulla et al. 2008; Wilkes et al. 2001; Waters et al. 2001; Song et al. 2015; Takil et al. 2002). There was a significant difference between groups, with MD − 2.26 mmol/L higher in the non-buffered group (95% CI - 2.84 to − 1.68; I^2^ = 56%;). Two trials with a total of 91 participants reported postoperative day one serum sodium of 140.6 mmol/L in the buffered fluid group and 141.8 mmol/L in the non-buffered fluid group (Kulla et al. 2008; Takil et al. 2002). There were no significant differences between groups, with serum sodium 1.2 mmol/L higher in the non-buffered fluid group (95% CI -2.55 to 0.12; *I*^2^ = 0).

##### Serum potassium

Seven trials with a total of 459 participants reported postoperative serum potassium. Potassium was 4.13 mmol/L in the buffered group and 4.22 mmol/L in the non-buffered group (Hadimioglu et al. 2008; Khajavi et al. 2008; Nuraei et al. 2010; O’Malley et al. 2005; Kulla et al. 2008; Wilkes et al. 2001; Song et al. 2015). There were no significant differences between groups, with MD − 0.04 mmol/L lower in the buffered group (95% CI − 0.14 to 0.06; *I*^2^ = 65%).

##### Serum chloride

Ten studies with a total of 530 participants reported postoperative serum chloride. Postoperative chloride was 107.5 mmol/L in the buffered fluid group and 114.3 mmol/L in the non-buffered fluid group at this time point (Hadimioglu et al. 2008; O’Malley et al. 2005; Kulla et al. 2008; Wilkes et al. 2001; Base et al. 2011; Waters et al. 2001; Scheingraber et al. 1999; Song et al. 2015; Takil et al. 2002; McFarlane and Lee 1994). Serum chloride was significantly higher in the non-buffered fluid group (95% CI − 10.17 to − 3.38) with a MD 6.77 mmol/L. Five studies, including 258 participants, reported a mean serum chloride on the first postoperative day. Serum chloride continued to be significantly elevated into the first post-operative day with of 105.7 mmol/L in the buffered fluid group and 114.4 mmol/L in the non-buffered fluid group (Hadimioglu et al. 2008; Kim et al. 2013; Kulla et al. 2008; Wilkes et al. 2001; Takil et al. 2002). Serum chloride was 8.48 mmol/L higher in the non-buffered fluid group (95% CI − 15.88 to − 1.08).

##### Serum glucose

Three trials reported postoperative serum glucose of 6.0 mmol/ L for both buffered and non-buffered groups. There was no difference between groups (95% CI − 0.29 to 0.29; *I*^2^ = 0%) (Wilkes et al. 2001; Waters et al. 2001; Chin et al. 2006).

##### Serum lactate

No difference between groups in terms of serum lactate was identified in four trials, with a total of 199 participants. Pooled data showed a mean serum lactate of 2.27 mmol/L in the buffered fluid group and 1.62 mmol/L in the non-buffered fluid group (MD 0.52 mmol/L higher in the buffered group; 95% CI − 0.04 to 1.08).

#### Serum measures of coagulation

Limited data was found on clotting factor concentrations and functional measures of coagulation in the included studies. Two studies with a total of 181 participants (Kulla et al. 2008; Gan et al. 1999) reported activated partial thromboplastin time (APTT), Factor VIII and von Willebrand factor (vWF). Only serum vWF concentration was found to be significantly different between groups, with a mean difference 31.4 IU/L lower in the buffered fluid group than in the non-buffered fluid group (95% CI − 47.7 to − 15.1; *I*^2^ = 0%). The clinical significance of this finding is unclear. One trial reported prothrombin time (PT) (Gan et al. 1999), which was not significantly different between groups. Three trials reported thromboelastographic (TEG) data (Martin et al. 2002; Gan et al. 1999; Song et al. 2015). Two studies reported postoperative TEG data graphically (Martin et al. 2002; Gan et al. 1999). Therefore, we did not subject this measure to meta-analysis.

##### Blood loss

Eleven trials of 576 participants reported absolute intraoperative blood loss in millilitres (Martin et al. 2002; Khajavi et al. 2008; O’Malley et al. 2005; Kulla et al. 2008; Gan et al. 1999; Wilkes et al. 2001; Scheingraber et al. 1999; Song et al. 2015; Takil et al. 2002; Walsh et al. 1983). Two studies reported estimated blood loss in millilitres/kilograms and could not be included in the analysis because they did not report patient weight (Base et al. 2011; McFarlane and Lee 1994). Reflective of varied types of surgery conducted, ranging from abdominal aortic aneurysm repair to day case surgery, clinical heterogeneity between these trials was high. Two trials reporting less than 400 ml of estimated blood loss (O’Malley et al. 2005; Walsh et al. 1983) and two trials reporting estimated blood loss of 2 L or more (Waters et al. 2001; Takil et al. 2002). These results made it unlikely that any analysis would yield a clinically significant result if the group was analysed as a whole.

We performed a subgroup analysis to attempt to reduce clinical heterogeneity by grouping trials with less than 1000 ml of blood loss and those with blood loss of 1000 ml or more. Trials reporting blood loss less than 1000 ml (five studies with 202 participants) reported no important differences between groups and showed mean difference in intraoperative blood loss that was 5.90 ml higher in the buffered group (95% CI − 45.18 to 56.99; *I*^2^ = 0%) (Khajavi et al. 2008; O’Malley et al. 2005; Kulla et al. 2008; Scheingraber et al. 1999; Walsh et al. 1983). Trials reporting blood loss of 1000 ml or more (six studies with 374 participants) also reported no important differences in blood loss between groups and showed mean difference in intraoperative blood loss that was 173 ml lower in the buffered group (95% CI − 438.8 to 92.7; *I*^2^ = 13%) (Martin et al. 2002; Gan et al. 1999; Wilkes et al. 2001; Waters et al. 2001; Song et al. 2015; Takil et al. 2002).

##### Transfusion requirement

Seven trials of 409 participants reported intraoperative red blood cells, platelet or fresh frozen plasma transfusion (Martin et al. 2002; O’Malley et al. 2005; Gan et al. 1999; Wilkes et al. 2001; Scheingraber et al. 1999; Takil et al. 2002. There was no significant difference in the quantity of any blood products transfused between individuals given buffered fluids and those given non-buffered solutions.

#### Post-operative hospital length of stay

Five trials with a total of 348 participants reported hospital length of stay (O’Malley et al. 2005; Gan et al. 1999; Base et al. 2011; Waters et al. 2001; Takil et al. 2002). Established formulae were used to numerically convert median (IQR) data to mean (± SD) (Hozo et al. 2005). There were no significant differences between groups, with MD in-hospital stay of 0.37 (95% CI − 0.72 to 1.47; *I*^2^ = 16%; favouring the non-buffered group).

#### Other

None of the included trials addressed the outcomes of cost or functional health status, cost or quality of life measures.

Biochemical outcomes from all studies are summarised in Tables 1 and 2. Extracted data from included studies is contained in a table in Additional file 2: Table S1. A summary of the primary and significant findings from a meta-analysis is included in Additional file 3: Table S2.

**Table 1:** Summary of differences in renal function outcomes between non-buffered and buffered fluid groups

Outcomes	Absolute effects		No. of participants (studies)	*p*
	Outcome with non-buffered fluid (mean)	Outcome with buffered fluid (mean; mean difference with 95% CI)		
Urine output—intraoperative	799 ml	872 ml (MD 6.1 ml higher; 95% CI − 128.4 to 140.6)	459 (8 RCTs)	0.93
Creatine change—postoperative	MD in creatinine was 7.0 μmol/L lower in the buffered fluids group; 95% CI − 27.4 to 13.5		113 (2 RCTs)	0.50
Creatine change—postoperative day 1	MD in creatinine was 4.9 μmol/L lower in the buffered fluids group; 95% CI − 5.9 to 15.8		113 (2 RCTs)	0.37
Creatinine—postoperative	Non-transplant patients		127 (2 RCTs)	0.59
	79.5 μmol/L	76.2 μmol/L (MD 2.38 μmol/L lower; 95% CI − 10.98 to 6.23)		
	Renal transplant patients		110 (1 RCT)	0.01
	460 μmol/L	530 μmol/L (MD 70 μmol/L higher; 95% CI 14.31 to 125.69)		
Creatinine—postoperative day 1	Non-transplant patients		61 (1 RCT)	0.44
	80 μmol/L	86 μmol/L (MD 6 μmol/L higher; 95% CI − 21.23 to 9.23)		
	Renal transplant patients		150 (2 RCTs)	0.74
	353.5 μmol/L	336 μmol/L (MD 12.26 μmol/L lower; 95% CI − 85.10 to 60.57)		
Creatinine—postoperative day 3	Renal transplant patients		301 (4 RCTs)	0.98
	168 μmol/L	173 μmol/L (MD 0.47 μmol/L higher; 95% CI − 30.12 to 29.19)

**Table 2:** Summary of differences in serum biochemistry between non-buffered and buffered fluid groups

Outcomes	Absolute effects		No. of participants (studies)	*p*
	Outcome with non-buffered fluid (mean)	Outcome with buffered fluid (mean; mean difference with 95% CI)		
PaCO2	35.0 mmHg	34.9 mmHg (MD 1 mmHg higher; 95% CI 0.15 to 1.94)	446 (7 RCTs)	0.02
Postoperative pH	7.32	7.38 (MD 0.05 units higher; 95% CI 0.04 to 0.07)	720 (12 RCTs)	< 0.0001
Base excess	− 5.0	− 1.65 (MD 3.5 units higher; 95% CI 2.6 to 4.4)	459 (9 RCTs)	< 0.0001
Serum bicarbonate	18.6 mmol/L	21.6 mmol/L (MD 3.14 mmol/L; 95% CI 2.3 to 4.0)	478 (8 RCTs)	< 0.0001
Serum sodium—postoperative	139.4 mmol/L	137.3 mmol/L (MD 0.52 mmol/ L higher; 95% CI − 2.8 to − 1.7)	447 (8 RCTs)	< 0.0001
Serum sodium—postoperative day 1	141.8 mmol/L	140.6 mmol/L (MD 0.52 mmol/ L higher; 95% CI − 2.8 to −1.7)	91 (2 RCTs)	0.07
Serum potassium	4.22 mmol/L	4.13 mmol/L (MD 0.04 mmol/ L lower; 95% CI − 0.14 to 0.06)	459 (7 RCTs)	0.43
Serum chloride—postoperative	114.3 mmol/L	107.5 mmol/L (MD 6.77 mmol/ L lower; 95% CI − 3.38 to −10.17)	530 (10 RCTs)	0.0001
Serum chloride—postoperative day 1	114.4 mmol/L	105.7 mmol/L (MD 8.48 mmol/ L lower; 95% CI − 15.88 to −1.08)	258 (5 RCTs)	0.02
Serum glucose	6.0 mmol/L	6.0 mmol/L (MD no difference; 95% CI − 0.29 to 0.29)	145 (3 RCTs)	0.99
Serum lactate	1.6 mmol/L	2.3 mmol/L (MD 0.52 mmol/ L higher; 95% CI − 0.04 to 1.1)	199 (4 RCTs)	0.067
Serum calcium	1.6 mmol/L	2.0 mmol/L	47 (1 RCT)	0.0001

## Discussion

The effects of intravenous fluid on clinical outcomes is a topic of major interest, which has been thoroughly explored in critical care patients but remains controversial in the peri-operative setting. This systematic review provides a comprehensive analysis of current data, demonstrating a paucity of high-quality trials with relevant patient-centred outcomes. It is surprising that the randomised controlled trial data available for meta-analysis is so small, relative to the millions of patients that receiving intravenous fluids during surgery each year. Our data shows that for three studies including 267 patients, the choice of perioperative fluid, either buffered or unbuffered did not result in a significant difference in mortality. The GRADE evidence for this outcome was rated as low. The overall combined mortality from these studies was low at 2%.

The analysis of secondary outcome measures from 18 different RCTs of 1096 participants suggests that intravenous fluids containing a physiological buffer are a safe alternative to saline-based fluids for adult patients undergoing surgery. We found limited evidence on the effects of fluid therapies on postoperative organ dysfunction, particularly on renal failure. For patients not undergoing renal transplantation, there were no differences in terms of renal insufficiency or surrogate markers of renal dysfunction (urine output and serum creatinine). However, there were differences in metabolic variables in post-operative pH, chloride concentration, base-deficits and serum bicarbonate, without significant changes in other electrolytes such as serum potassium and sodium concentrations. High serum chloride is a cause of metabolic acidosis and may explain our findings of both lower pH and lower partial pressure of arterial carbon dioxide (PaCO_2_) (secondary to respiratory compensation for metabolic acidosis) when non-buffered fluids were used. We rated GRADE evidence for these secondary outcomes as low-moderate quality.

This review focused on the type of perioperative fluid administration. Evidence from studies investigating the volume of fluid and haemodynamic monitoring suggest that decisions regarding perioperative fluid strategy can influence outcome after surgery. Hence, it is not just the type of fluid that is important, but exactly how the fluid is administered, in terms of volume and timing. Protocols for fluid administration in the included studies in this review were seldom available. Studies of goal-directed fluid therapy offer supportive evidence for reduced morbidity and length of hospital stay (Pearse et al. 2014), but poor evidence for reductions in mortality (Calvo-Vecino et al. 2018). Excessive volumes of fluid administration have been associated with harm, such as pulmonary complications and tissue oedema. However, the current trend for conservative fluid administration, as advocated in many perioperative enhanced recovery pathways, has also been recently disputed a major trial measuring an increased proportion of acute kidney injury in patients who received a zero fluid balance regimen in the perioperative period (Myles et al. 2018). In the face of conflicting evidence, getting the fluid strategy right is not easy. However, it is becoming clear that an inflexible, “one size fits all” approach when planning perioperative fluid management may not be the best approach, and a strategy that has been individualised to each patient may be more appropriate with many variable factors taken into consideration. This is reflected in fluid management consensus statements such as the recent American Society of Enhanced Recovery and Perioperative Quality Initiative (POQI) joint statement that advocates an individualised approach to fluid management, taking into account patient-specific variables (Thiele et al. 2016).

In addition to haemodynamic optimisation and total volume dosing, fluid electrolyte composition is also important to consider. Perceived advantages of balanced crystalloid solutions over non-buffered solutions are reflected in the British Consensus Guidelines on Intravenous Fluid Therapy for Adult Surgical Patients (GIFTASUP) (Powell-Tuck et al. 2011) that recommend the use of balanced solutions for crystalloid fluid resuscitation or replacement.

Our findings are consistent with a recent systematic review and meta-analysis, which included studies from ICU patients and demonstrated no difference in the outcomes of hospital mortality, the occurrence of acute kidney injury or need for renal replacement therapy with balanced intravenous fluid resuscitation (Neto et al. 2017). A large single centre, pragmatic, crossover trial comparing lactated Ringer’s solution or Plasma-Lyte A with saline in emergency admissions of non-critically ill adults was published recently. This study, whilst not exclusively conducted in peri-operative patients, included about 20% of general surgical patients and concluded that there was no significant between-group difference in mortality or hospital-free days at day 28. However, the incidence of major adverse kidney events, within 30 days, was lower with balanced fluids (4.7%), compared with saline (5.6%) (Self et al. 2018). A similar trial design was adopted by a multi-centre study of > 15,000 critically ill patients comparing saline with buffered crystalloids (lactated Ringer’s solution or Plasma-Lyte A) that reported a reduction of 1.1% in the composite primary outcome of major adverse kidney events within 30 days (Semler et al. 2018). In both of these studies, the primary outcome of major adverse kidney event within 30 days was defined as a composite of death, new-renal replacement therapy or persistent renal dysfunction (final serum creatinine concentration of > 200% of the baseline). Whilst these large pragmatic studies are highly informative, they were open-label trials reporting a composite outcome, which can be associated with potential caveats of multiple sources of bias and are not specific to surgical patients.

A further, recent study was published subsequent to the search strategy for this review and is therefore not included in the meta-analysis. This double-blind randomised trial of saline versus balanced crystalloid for goal-directed perioperative fluid therapy in major abdominal surgery patients was terminated for safety reasons after recruiting 60 patients of a planned sample size of 240 (Pfortmueller et al. 2018). Patients in the saline group developed hyperchloraemic metabolic acidosis, but also a dose-dependent increase in vasopressor requirements, despite no difference in total in total perioperative fluid volumes. This study signals a serious measure of harm, albeit in an underpowered study format, for perioperative saline administration.

Whilst we used an inclusive search strategy and eligibility criteria to maximise identification of relevant studies, this meant that there was considerable clinical heterogeneity in participant characteristics, types of surgery and protocols for administering fluids in the trials. Some RCTs involved minor surgery in otherwise healthy patients (Chin et al. 2006), whilst others analysed outcomes after very major surgery in high-risk patient groups (Wilkes et al. 2001; Waters et al. 2001). This is important because both the baseline values and tolerance of the magnitude of homeostatic derangement will vary with patient organ function, meaning that a single recommendation cannot be generalised to the whole patient population. For example, an observation that fluid choice influences renal failure in patients with pre-existing severe renal dysfunction is unlikely to be applicable to patients with normal glomerular filtration rates. Subgroup analysis and reporting of multiple secondary outcomes was conducted in order to clarify the results from pooled data. Only trials included adult perioperative patients were included, hence conclusions cannot be directly drawn for paediatric or medically unwell patients from this meta-analysis. Whilst statistical heterogeneity for our primary outcome of mortality was very low (*I*^2^ = 0), many of our secondary outcomes displayed substantially greater statistical heterogeneity.

Some trial data did not contribute to our analyses because they were reported in weight-based units rather than in absolute amounts. We attempted to contact trial authors to obtain individual participant data, but we were not always successful in these attempts.

This review identified small numbers of patients and low numbers of events across outcomes of interest, including the primary outcome of mortality. Alongside this several of the studies were poorly reported and at high or unclear risk of bias. Where relevant, the consequent downgrading of the quality of evidence reduces the confidence in reported effects in pooled data.

Although there are a substantial number of studies in this systematic review, no single, large pragmatic and appropriately blinded randomised controlled trial was identified with sufficient power to detect important differences in clinical outcomes arising from the choice of perioperative fluid. In particular, acute kidney injury should be considered as an important and patient-relevant outcome measure. The SOLAR fluid trial, a large study of saline versus Ringer’s lactate that is assessing major postoperative complications as its primary outcome measure, is currently ongoing and expected to be completed in 2022 (SOLAR trial 2017).

Buffered and non-buffered fluids have predictable effects on post-operative biochemical parameters in surgical patients, are appropriate for fluid replacement and should be considered especially for patients with, or at risk of, metabolic derangement. No complications or adverse effects specific to buffered fluids were identified in surgical patients with a range of co-morbidities and organ dysfunction.

Additional studies are needed, including well designed and adequately powered randomised controlled trials to detect differences in clinical outcomes arising from the physician’s choice of perioperative fluid. Such studies should include meaningful patient-centred outcomes such as mortality, quality of recovery, length of hospital stay, and organ dysfunction (including acute kidney injury) and quality of life measures such as postoperative pain.

## Additional files

Additional file 1: Search strategies for CENTRAL, MEDLINE, Embase and CINHAL. (DOCX 16 kb)

Additional file 2: **Table S1.** Characteristics of included studies. (DOCX 49 kb)

Additional file 3: **Table S2.** Summary of findings for the main comparison. (DOCX 16 kb)

## Abbreviations

CI: Confidence intervals
GIFTASUP: British Consensus Guidelines on Intravenous Fluid Therapy for Adult Surgical Patients
HES: Hydroxyethyl starch
MD: Mean difference
MW: Molecular weight
OR: Odds ratio
RCT: Randomised controlled trial
