# Supplementary material for: Perioperative administration of buffered versus non-buffered crystalloid intravenous fluid to improve outcomes following adult surgical procedures: a Cochrane systematic review
Source: Perioper Med (Lond). 2018 Dec 13;7:27. doi: 10.1186/s13741-018-0108-5 (PMC6291967; doi:10.1186/s13741-018-0108-5)
Supplement: Supplementary file 1 — Search strategies for CENTRAL, MEDLINE, Embase and CINHAL. (DOCX 16 kb) [file 13741_2018_108_MOESM1_ESM.docx]

**Additional file 1: Search strategies for CENTRAL, MEDLINE, Embase and CINHAL**

**Search strategy for CENTRAL, the Cochrane Library**

#1 MeSH descriptor Colloids, this term only

#2 colloid*:ti,ab

#3 crystalloid*

#4 MeSH descriptor Plasma Substitutes, this term only

#5 (lactated or colloid* or hyperchlor?emi* or crystalloid* or ringer or hartmann or “Fluid Therapy” or buffered):ti,ab #6 (fluid* near (intravenous or replacement or resuscitation or balanced or non-balanced)):ti,ab

#7 saline:ti

#8 (#1 OR #2 OR #3 OR #4 OR #5 OR #6 OR #7) #9 MeSH descriptor Surgery explode all trees

#10 (surgery or surgical):ab

#11 (#9 OR #10)

#12 (#8 AND #11)

**Search strategy for MEDLINE (OvidSP)**

1. colloid*.ti,ab. or Colloids/

2. crystalloid*.mp.

3. lactated.ti,ab. or Plasma Substitutes/

4. (hyperchlor?emi* or crystalloid* or ringer or hartmann).ti,ab.

5. Fluid Therapy.ti,ab. or Fluid Therapy/

6. (intravenous adj3 fluid*).ti,ab.

7. (fluid adj3 replacement).mp.

8. (fluid and resuscitation).ti,ab.

9. buffered.mp. or exp Bicarbonates/

10. ((balanced or non-balanced) adj3 fluid*).mp.

11. saline.ti.

12. 7 or 5 or8 or 1 or 6 or 2 or 10 or 4 or 3 or 11 or 9

13. Surgery/ or (surgery or surgical*).ti,ab.

14. 13 and 12

15. ((randomised controlled trial or controlled clinical trial).pt. or randomized.ab. or placebo.ab. or clinical trials as topic.sh. or randomly.ab. or trial.ti.) and humans.sh.

16. 15 and 14

**Search strategy for Embase (OvidSP)**

1. colloid*.ti,ab. or Colloids/

2. crystalloid*.mp.

3. actated.ti,ab. or Plasma Substitutes/

4. (hyperchlor?emi* or crystalloid* or ringer or hartmann).ti,ab. 5. Fluid Therapy.ti,ab. or Fluid Therapy/

6. (intravenous adj3 fluid*).ti,ab.

7. (fluid adj3 replacement).mp.

8. (fluid and resuscitation).ti,ab.

9. buffered.mp. or exp Bicarbonates/

10. ((balanced or non-balanced) adj3 fluid*).mp.

11. saline.ti.

12. 1 or 2 or 3 or 4 or 5 or 6 or 7 or 8 or 9 or 10 or 11

13. Surgery/ or (surgery or surgical*).ti,ab.

14. 13 and 12

15. (placebo.sh. or controlled study.ab. or random*.ti,ab. or trial*.ti,ab.) and human*.ec,hw,fs. 16. 15 and 14

**Search strategy for CINAHL (EBSCOhost)**

#1. TX (Fluid Therapy)

#2. (resuscitation or replacement or intravenous or balanced or non balanced) and fluid

#3. TX buffered or colloid* or crystalloid* or acetated or hyperchloremi* or hyperchloraemi* or crystalloid* or ringer or hartmann #4. TI saline

#5. MW Bicarbonates or Fluid Therapy or Plasma Substitutes or Colloids

#6. #1 or #2 or #3 or #4 or #5

#7. TX surgery or surgical

#8. #6 and #7

#9. (MH “Clinical Trials+”)

#10. (“randomised”) or (MM “Random Assignment”)

#11. #9 or #10

#12. #8 and #11
