# Supplementary material for: Perioperative administration of buffered versus non-buffered crystalloid intravenous fluid to improve outcomes following adult surgical procedures: a Cochrane systematic review
Source: Perioper Med (Lond). 2018 Dec 13;7:27. doi: 10.1186/s13741-018-0108-5 (PMC6291967; doi:10.1186/s13741-018-0108-5)
Supplement: Supplementary file 2 — Table S1. Characteristics of included studies. (DOCX 49 kb) [file 13741_2018_108_MOESM2_ESM.docx]

Additional file 2: **Table S1** Characteristics of included studies

| **Author** | **Methods** | **Participants** | **Surgery** | **Intervention** | **Outcomes** | **Notes** |
| --- | --- | --- | --- | --- | --- | --- |
| **Base 2011** | RCT  No withdrawals  2 European cardiac surgery hospitals  Sample size =  81 participants | Age 67 yo  62M/19F  ASA not reported | CABG, valve surgery  4h duration | **Buffered n = 43**  Balanced 6% HES 130/0.4 (Volulyte) for intraoperative and postoperative fluid administration  **Control n = 38**  6% HES 130/0.4 in saline for intraoperative and postoperative fluid administration | Acid-base status, serum biochemistry up to 24 hours postoperatively. Duration of ICU stay, duration of hospital stay, and mortality all measured up to 30 days postoperatively |  |
| **Chin 2006** | RCT  10 withdrawals  Single hospital in Singapore  Sample size =  50 participants | Age 45 yo  21M/11F  ASA I or II | Elective surgery that was not expected to enter into a major body cavity, or to require IV fluid volume >500 mL in the first 2 hours of perioperative care. Covered orthopaedic, ENT, breast, minor general surgery  1.4h duration | **Buffered n = 16**  500 mL lactated Ringer’s solution administered over 45 to 60 minutes  **Control n = 38**  500 mL 0.9% normal saline administered over 45 to 60 minutes | Serum glucose and electrolytes, measured up to 1 hour postoperatively | One arm consisted of participants who received a fluid formulation that was not relevant to this review (dextrose 5% in 0.9% saline). Details for this arm of the study are not extracted here. The study included only minor surgery, with patients who received only a small amount of intravenous fluid. |
| **Gan 1999** | RCT  3 withdrawals  2 hospitals in USA  Sample size = 120 | Age 58 yo  69M/61F  ASA I to II | major elective surgery, which covered orthopaedic, general, gynaecological, and urological needs  5.3h duration | **Buffered n = 60**  Hextend administered via a hypovolaemia algorithm to ensure adequate volume during the operation  **Control n = 38**  Hespan administered via a hypovolaemia algorithm to ensure ade- quate volume during the operation  Each arm was given a maintenance dose of lactated Ringer’s solution (a buffered fluid) | Urine output, EBL, intraoperative transfusion, death, length of postoperative stay, re- quirement for calcium measured and recorded during hospital stay |  |
| **Hadimioglu 2008** | RCT  Double blinded  Withdrawals not reported  Single or multicentre not reported  Sample size = 90 | Age 46 yo  M/F not specificed  ASA III to IV with end stage renal failure | living-related kidney transplants  2h duration | **Buffered n = 60**  30 patients received Lactated Ringer’s  30 patients received Plasmaltye 148  **Control n = 38**  0.9% saline | Urine output, pH, base excess, lactate, bicarbonate, potassium, CO2 , chloride, creatinine, creatinine clearance, chloride, requirement for dialysis measured daily until the third postoperative day and then once more on the seventh postoperative day | The 2 buffered arms (lactated Ringer’s solution and Plasmalyte 148) were numerically combined for meta-analysis, so that the buffered fluid arm included 60 participants |
| **Heidari 2011** | RCT  Double blinded  No withdrawals  Single centre in Middle East  Sample size = 90 | Age 42 yo  28M/32F  ASA I to II | elective lower abdominal surgery  1.4h duration | **Buffered n = 60**  Ringer’s lactate 15 mL/kg administered 30 minutes preoperatively  **Control n = 38**  normal saline 15 mL/kg administered 30 minutes preoperatively | Postoperative nausea using VAS and incidence of postoperative vomiting at 6, 12, and 24 hours postoperatively | One arm consisted of participants who received a fluid formulation that was not relevant to this review. Details for this arm of the study are not extracted here. |
| **Khajavi 2008** | RCT  Double blinded  Single centre in Middle East  Sample size = 52 | Age 38 yo  Gender not described in detail, but groups described as sex-matched ASA not reported, but assumed III to IV, given end stage renal failure | elective renal transplantation surgery  Surgery duration not described | **Buffered n = 26**  Ringer’s lactate 60 mL/kg titrated to a CVP of 10 to 15 mmHg  **Control n = 26**  normal saline 60 mL/kg titrated to a CVP of 10 to 15 mmHg | Electrolytes, pH, blood loss, graft failure, urine output Blood samples measured at the start of surgery, at 1 hour, and at end of surgery. Urine output measured first 4 hours postoperatively |  |
| **Kim 2013** | RCT  Double blinded  Single centre in Korea  Sample size = 60 | Age 47 yo  38M/22F  ASA not reported, but assumed III to IV, given end stage renal failure | elective renal transplantation surgery  4.4h duration | **Buffered n = 30**  Ringer’s lactate infusion to maintain CVP of 12 to 15 mmHg  **Control n = 30**  normal saline infusion to maintain CVP of 12 to 15 mmHg | pH, base excess, strong ion difference, urine output, postoperative creatinine, graft failure requiring dialysis up to day 7 postoperatively |  |
| **Kulla 2008** | RCT  Double blinded  Single centre in Germany  Sample size = 62 | Age only described as adult  Gender not reported  ASA II to III | elective major abdominal surgery  Surgery duration not reported | **Buffered n = 29**  buffered HES as colloid and acetated Ringer’s solution  **Control n = 33**  Non-buffered arm - HES in saline-based solution as colloid plus a non-balanced crystalloid | Endpoints taken preoperatively, during surgery, at end of surgery, 6 hours postoperatively, and at POD1 blood loss, urine output, creatinine, lactate, sodium, chloride, PaO2, PaCO2, pH, base excess, thromboplastin time “quick”, partial thromboplastin time, antithrombin III, Factor VIII, von Willebrand factor, ristocetin cofactor | Very heterogeneous group of surgical procedures, including 1 each of oesophagectomy, gastrectomy, prostatectomy, nephrectomy, and colonic surgery |
| **Martin 2002** | RCT  No withdrawals  Single centre in USA  Sample size = 90 | Age 58 yo  Gender not reported  ASA I to III | major elective non-cardiac surgery with anticipated blood loss of 500 mL  Surgery duration not reported | **Buffered n = 30**  Hextend, given to ensure adequate volume resuscitation during surgery  **Control n = 30**  Hespan, given to ensure adequate volume resuscitation during surgery  In addition, each arm was given a maintenance dose of lactated Ringer’s solution (a buffered fluid) | TEG data measured before induction, at end of surgical procedure, and 24 hours post- operatively | These are the same participant data as in Moretti 2003, but different outcomes are reported.  One arm consisted of participants who received a fluid formulation that was not relevant to this review. Details for this arm of the study are not extracted here. |
| **McFarlane 1994** | RCT  No withdrawals  Single centre in UK  Sample size = 30 | Age 58 yo  Gender not reported  ASA I to II | major elective hepatobiliary or pancreatic surgery  3.5h duration | **Buffered n = 15**  Plasmalyte 148 infusion 15 mL/kg/h  **Control n = 15**  normal saline infusion 15 mL/kg/h  In addition all fluids were warmed and rate of administration was adjusted in response to clinical state, but not according to a defined protocol | Blood loss (mL/kg) mean and SD, chloride, bicarbonate, base excess, PaCO2, lactate up to 24 hours postoperatively |  |
| **Moretti 2003** | RCT  No withdrawals  Single centre in USA  Sample size = 90 | Age 58 yo  Gender not reported  ASA I to III | major elective non-cardiac surgery with anticipated blood loss of 500 mL  Surgery duration not reported | **Buffered n = 30**  Hextend, given to ensure adequate volume resuscitation during surgery  **Control n = 30**  Hespan, given to ensure adequate volume resuscitation during surgery  In addition, each arm was given a maintenance dose of lactated Ringer’s solution (a buffered fluid) | Postoperative nausea and vomiting, oedema up to 24 hours after surgery | These are the same participant data as in Martin 2002, but different outcomes are reported.  One arm consisted of participants who received a fluid formulation that was not relevant to this review. Details for this arm of the study are not extracted here. |
| **Nuraei 2010** | RCT  No withdrawals  Single centre in Iran  Sample size = 108 | Age 38yo  68M/40F  ASA reported as I and II, but all participants had end-stage renal disease and were awaiting transplant | renal transplant surgery  2h duration | **Buffered n = 54**  Ringer’s lactate  **Control n = 54**  Normal saline  Protocol for fluid administration not reported | Renal function, acid-base status up to 24 hours after surgery |  |
| **O’Malley 2005** | RCT  Double blinded  3 withdrawals due to high pre-operative potassium  Single centre in USA  Sample size = 54 | Age 44 yo  32M/19F  ASA >III | renal transplantation  5.6h duration | **Buffered n = 25**  Ringer’s lactate  **Control n = 26**  Normal saline  Fluids titrated to routine clinical endpoints IV dopamine infusion 2 mcg/kg/min to all patients | Serum potassium, pH, serum creatinine up to 1 week, postoperative urine output, creatinine clearance, requirement for dialysis, blood loss, transfusion requirements, length of stay in hospital | Trial was stopped early when severe hyperkalaemia was noted in some participants in the control arm |
| **Scheingraber 1999** | RCT  Double blinded  No withdrawals  Single centre in USA  Sample size = 24 | 46F (no male patients)  ASA I to II | elective gynaecological surgery  2.3h duration | **Buffered n = 12**  Ringer’s lactate  **Control n = 12**  Normal saline  Supplemental intravenous potassium administered according to intra-operative serum potassium levels. During the study, no participants received colloids, plasma products, or blood transfusions. | Estimated intraoperative blood loss, urine output, pH, carbon dioxide, base excess, lactate, chloride, sodium |  |
| **Song 2015** | RCT  Double blinded  No withdrawals  Single centre in Korea  Sample size = 50 | Age 44 yo  32M/19F  ASA >III | elective lumbar spinal surgery  4.9h duration | **Buffered n = 25**  Ringer’s lactate  **Control n = 25**  Normal saline  Fluids administered at 6 mL/kg/h; increased at discretion of anaesthetist. If blood loss > 500 mL, participants were given a colloid (6% HES in saline). If haematocrit decreased to < 24%, blood was given. No significant differences between groups in volumes of blood or colloid administered. | Rotational thromboelastometry (ROTEM) at end of surgery. Intraoperative blood loss, pH, BE, bicarbonate. Postoperative electrolytes at 12 hours Urea and creatinine at 24 hours |  |
| **Takil 2002** | RCT  Double blinded  No withdrawals  Single centre in Turkey  Sample size = 30 | Age 41 yo  Gender not reported  ASA I to II | elective major spinal surgery  4.9h duration | **Buffered n = 15**  Ringer’s lactate  **Control n = 15**  Normal saline  Fluids administered at 20 mL/kg/h during surgery. If blood loss > 500 mL, participants were given a colloid (Gelofusine) . If blood loss > 20%, blood was given. | Sodium, chloride, bicarbonate, base deficit, pH, blood transfusion, blood loss, urine output, PaCO2 up to 12 hours after surgery. Length of intensive care unit and hospital stay recorded Time points were first, second, fourth, sixth, and twelfth hours postoperatively |  |
| **Walsh 1983** | RCT  No withdrawals  Single centre in UK  Sample size = 21 | Age 52 yo  Gender not reported  ASA not reported | elective cholecystectomy  Surgery duration not reported | **Buffered n = 7**  Ringer’s lactate  **Control n = 7**  Normal saline  Fluids administered at 6 mL/kg/h during surgery | Estimated blood loss measured intraoperatively | 3 arms included in the trial, with 7 participants in each. Arms were given lactated Ringer’s solution, 5% dextrose, and normal saline. Only 2 arms were included for analysis (N = 14) |
| **Waters 2001** | RCT  No withdrawals  Single centre in USA  Sample size = 66 | Age 70 yo  Gender not reported  ASA III | open abdominal aortic aneurysm surgery  Surgery duration not reported | **Buffered n = 33**  Ringer’s lactate  **Control n = 33**  Normal saline  Fluids administered to maintain CVP to within 10% of baseline. Colloid administration restricted to period of rapid blood loss. Protocol allowed sodium bicarbonate to be given to participants if their metabolic acidosis was significant. Participants were given human albumin solution in addition to study fluid at the discretion of the anaesthetic team. All cell-salvaged blood was washed in normal saline. | Urine output, creatinine, need for renal replacement therapy, EBL, transfusion requirements, base deficit, chloride, death, length of postoperative stay Variables were measured at start of surgery, on admission to ICU, and every 24 hours until normalization of measured variable | All patients received thoracic epidural. Non-buffered arm received an average of 1500 mL more fluid intraoperatively |
| **Wilkes 2001** | RCT  Double blinded  No withdrawals  2 centres in UK  Sample size = 47 | Age 72 yo  23M/24F  ASA I to III | major non-cardiac surgery  3.2h duration | **Buffered n = 23**  Hartmann’s and 6% Hetastarch  **Control n = 24**  normal saline and 6% Hetastarch  500 mL of colloid at induction as a bolus followed by 7 mL/kg/h of crystalloid as an infusion according to a predefined algorithmic protocol. | Chloride, sodium, RBCs transfused, platelets transfused, FFP transfused, urine output, base excess, pH, PaCO2, bicarbonate measured postoperatively | Trial was stopped early after 1 participant experienced adverse effects that may have been caused by the study fluid |

NB. Age and surgery duration are means
