# Supplementary material for: Perioperative administration of buffered versus non-buffered crystalloid intravenous fluid to improve outcomes following adult surgical procedures: a Cochrane systematic review
Source: Perioper Med (Lond). 2018 Dec 13;7:27. doi: 10.1186/s13741-018-0108-5 (PMC6291967; doi:10.1186/s13741-018-0108-5)
Supplement: Supplementary file 3 — Table S2. Summary of findings for the main comparison. (DOCX 16 kb) [file 13741_2018_108_MOESM3_ESM.docx]

Additional file 3: **Table S2** Summary of findings for the main comparison

Buffered versus non-buffered crystalloid intravenous fluid for adults undergoing any form of surgery

Patient or population: adults receiving intravenous fluids whilst undergoing any form of surgery Setting: elective, major surgery in hospitals in Europe, USA, Asia, and the Middle East

Intervention: buffered crystalloid intravenous fluid

Comparison: non-buffered crystalloid intravenous fluid

| **Outcomes** | **Anticipated absolute effects (95% CI)** | | **Relative effect (95% CI)** | **No. of participants (studies)** | **Quality of the evidence (GRADE)** |
| --- | --- | --- | --- | --- | --- |
|  | **Risk with non-buffered fluid** | **Risk with buffered fluid** |  |  |  |
| **Mortality (in-hospital death and 30-day mortality)** | Study population | | OR 1.85 (0.37 to 9.33) | 267 (3 RCTs | Low |
|  | 15 per 1000 | 28 per 1000 (6 to 126) |  |  |  |
| **Organ system failure - renal insufficiency requiring support** | Study population | | OR 0.82 (0.34 to 1.98) | 267 (4 RCTs) | Low |
|  | 92 per 1000 | 77 per 1000 (33 to 168) |  |  |  |
| **Plasma pH - postoperative pH** | Mean postoperative pH was 7.32 | Mean postoperative pH in intervention group was 0.05 higher (0.04 to 0.07) | - | 720 (12 RCTs) | Moderate |
| **Serum chloride (mmol/ L) - postoperative chloride** | Mean postoperative chloride was 114.3 mmol/L | Mean postoperative chloride in intervention group was 6.77 mmol/ L lower (3.38 to 10.17) | - | 530 (10 RCTs) | Moderate |
